# Cardiac structure and function in relation to cardiovascular risk factors in Chinese

**DOI:** 10.1186/1471-2261-12-86

**Published:** 2012-10-04

**Authors:** Yi Zhang, Yan Li, Ming Liu, Chang-Sheng Sheng, Qi-Fang Huang, Ji-Guang Wang

**Affiliations:** 1 Centre for Epidemiological Studies and Clinical Trials, The Shanghai Institute of Hypertension, Ruijin Hospital, Shanghai Jiaotong University School of Medicine, Ruijin 2nd Road, Shanghai 200025, China

**Keywords:** Left atrial enlargement, Left ventricular hypertrophy, Left ventricular diastolic dysfunction, Blood pressure

## Abstract

**Background:**

Cardiac structure and function are well-studied in Western countries. However, epidemiological data is still scarce in China.

**Methods:**

Our study was conducted in the framework of cardiovascular health examinations for the current and retired employees of a factory and their family members. According to the American Society of Echocardiography recommendations, we performed echocardiography to evaluate cardiac structure and function, including left atrial volume, left ventricular hypertrophy and diastolic dysfunction.

**Results:**

The 843 participants (43.0 years) included 288 (34.2%) women, and 191 (22.7%) hypertensive patients, of whom 82 (42.9%) took antihypertensive drugs. The prevalence of left atrial enlargement, left ventricular hypertrophy and concentric remodeling was 2.4%, 5.0% and 12.7%, respectively. The prevalence of mild and moderate-to-severe left ventricular diastolic dysfunction was 14.2% and 3.3%, respectively. The prevalence of these cardiac abnormalities significantly (*P* ≤ 0.002) increased with age, except for the moderate-to-severe left ventricular diastolic dysfunction. After adjustment for age, gender, body height and body weight, left atrial enlargement was associated with plasma glucose (*P* = 0.009), and left ventricular hypertrophy and diastolic dysfunction were significantly associated with systolic and diastolic blood pressure (*P* ≤ 0.03), respectively.

**Conclusions:**

The prevalence of cardiac structural and functional abnormalities increased with age in this Chinese population. Current drinking and plasma glucose had an impact on left atrial enlargement, whereas systolic and diastolic blood pressures were major correlates for left ventricular hypertrophy and diastolic dysfunction, respectively.

## Background

Cardiac structure and function are well-studied in Western populations, such as the Framingham Heart Study [1,2], the Rotterdam Study [3], and the Atherosclerotic Risk in Communities (ARIC) Study [4]. Cardiac structural and functional abnormalities, even if at asymptomatic stage, may predict cardiovascular events and mortality [1-8]. In the Framingham Heart Study, the echocardiographically diagnosed left ventricular hypertrophy was associated with a 73% and 112% higher risk of cardiovascular mortality, and a 49% and 101% higher risk of all-cause mortality in men and women, respectively [1].

Several clinical studies reported prevalence and correlates of left ventricular hypertrophy in Chinese hypertensive patients [9,10]. However, there is limited epidemiological data on cardiac structural and functional abnormalities in Chinese population. We therefore performed echocardiographic measurements in the framework of cardiovascular health examinations for employees of a factory located in southeast China. In the present cross-sectional analyses, we investigated the prevalence and correlates of cardiac structural and functional abnormalities, including left atrial enlargement, left ventricular hypertrophy and diastolic dysfunction.

## Methods

### Study participants

The present study was conducted in the year 2006 in the framework of comprehensive cardiovascular health examinations for all employees (including those retired but still lived in the area of the factory at the time of the study) of a factory located in an isolated coast area, 300 kilometers south of Shanghai [11]. The family members of these employees at least 12 years of age living in the nearby region were also invited for participation. The Ethics Committee of Ruijin Hospital, Shanghai Jiaotong University School of Medicine approved the study protocol. All subjects gave written informed consent.

Of the 1052 study subjects (participation rate 80.7%), 877 participated in the echocardiographic study. We excluded 34 subjects from the present analysis, because they had poor quality of echocardiogram (n = 9), moderate or severe valvular regurgitation or stenosis (n = 20), congenital heart disease (n = 3), atrial fibrillation (n = 1) or hypertrophic cardiomyopathy (n = 1). Thus, the number of participants included in the present analysis was 843.

### Field work

One experienced physician measured each participant’s blood pressure five times consecutively by conventional sphygmomanometry, after at least 5 minutes rest in the sitting position. These five blood pressure readings were averaged for analyses. The same observer also administered a standardized questionnaire to collect information on medical history, smoking habits, alcohol consumption and the use of medications. Current smoking and drinking were defined as at least smoking one cigarette per day or drinking once per week in the past year, respectively. Daily alcohol consumption was calculated according to the consumption of various types of alcoholic beverages during the past month. In the study area, beer was sold in a bottle of 750 mL, whereas yellow aperitif, rice aperitif and hard liquor were traded in a unit of 500 grams. We estimated that one bottle of beer and a unit of aperitif and liquor contained on average 30, 90 and 200 g of ethanol, respectively. From the type and quantity of the alcoholic beverages used, we computed daily alcohol consumption in grams per day. Drinking subjects were further categorized into tertiles of daily alcohol consumption, with mild, moderate and heavy drinking defined as daily alcohol consumption <18 g/d, 18 to 36 g/d, and > 36 g/d, respectively. Hypertension was defined as a blood pressure of at least 140 mmHg systolic or 90 mmHg diastolic, or the use of antihypertensive drugs. One trained technician measured body height and body weight. Body mass index was calculated as body weight in kg divided by the square of body height in meters. Venous blood samples were taken after overnight fasting for measurements of plasma glucose concentration and concentrations of total, high-density lipoprotein (HDL), and low-density lipoprotein (LDL) cholesterol and triglycerides.

### Echocardiographic measurements

One echocardiographer performed all echocardiographic measurements using the portable Mylab 30 CV machine (ESAOTE SpA, Genoa, Italy) according to the American Society of Echocardiography (ASE) recommendations. Two-dimensional and color Doppler images were first recorded, and then analyzed offline. Moderate or severe valvular regurgitation or stenosis was diagnosed as valvular heart disease using color Doppler imaging [12].

Left atrial volume was calculated using the ellipse model formula: left atrial volume = π × (SA1 × SA2 × LA)/6, where SA1 is the M-mode left atrial dimension in the parasternal short-axis view and SA2 and LA are measurements of short- and long-axes in the apical four chamber view at ventricular end-systole [13]. Left atrial volume was standardized to body size, as left atrial volume index. Left atrial enlargement was defined as left atrial volume index ≥ 29 mL/m^2^ according to ASE recommendations [14].

Measurements for M-mode guided calculation of left ventricular mass (LVM) were taken in the parasternal short-axis view. Left ventricular internal dimension at end-diastole (LVIDd) and septal (SWTd) and posterior wall thickness at end-diastole (PWTd) were measured. LVM was calculated by the ASE recommended formula [15]: LVM (g) = 0.8 × {1.04 × [(LVIDd + PWTd + SWTd)^3^ - (LVIDd)^3^} + 0.6, and standardized to body size by dividing the raw LVM by body surface area, as left ventricular mass index (LVMI). Relative wall thickness (RWT) was calculated by the formula: (2 × PWTd)/LVIDd, and also used as a measure of left ventricular geometry [14]. Left ventricular concentric remodeling and eccentric and concentric hypertrophy were defined as RWT > 0.42 and LVMI ≤ 115 g/m2 (men) or LVMI ≤ 95 g/m2 (women), RWT > 0.42 and LVMI > 115 g/m2 (men) or LVMI > 95 g/m2 (women), and RWT < 0.42 and LVMI > 115 g/m2 (men) or LVMI > 95 g/m2 (women), respectively [15].

Mitral and pulmonary venous inflow (pulse-wave Doppler) and left atrial volume index were measured for the diagnosis of left ventricular diastolic dysfunction. Normal, indeterminate, and mild, moderate and severe left ventricular diastolic dysfunction were further classified according to the ASE recommendations [16]. On the basis of the ratio of early transmitral diastolic peak flow (E) and atrial peak flow (A, E/A ratio), and the deceleration time of early transmitral diastolic flow (DTE), all subjects were categorized into 3 groups: category 1 with an E/A ratio <0.75 and a prolonged DTE (>220 ms), category 2 with normal range of E/A ratio (0.75 to 1.5) and DTE >150 ms, and category 3 with E/A ratio >1.5 and DTE < 150 ms. Category 1 was diagnosed as mild left ventricular diastolic dysfunction. Category 2 was further differentiated as normal, indeterminate or moderate left ventricular diastolic dysfunction, if none, only one or at least 2 of the following 4 abnormalities were respectively present: pulmonary venous systolic velocity less than diastolic velocity (S/D < 1); pulmonary venous atrial reversal duration greater than mitral A duration more than 30 ms (ARD-AD > 30 ms); peak of pulmonary venous atrial reversal wave more than 35 cm/s (AV > 35 m/s); and left atrial volume index more than 29 ml/m^2^. Category 3 was further differentiated as indeterminate or severe left ventricular diastolic dysfunction in the absence or presence of at least 1 of the 4 above-mentioned abnormalities. Only in the presence of all echocardiographic measurements mentioned above, left ventricular diastolic function could be evaluated, so that 150 subjects were excluded, leaving 693 subjects in the analysis of diastolic dysfunction.

Left ventricular ejection fraction (EF) was measured by quantitative two-dimensional method (biplane Simpson method) [14].

### Statistical methods

Statistical analysis was performed using SAS software, version 9.1 (SAS Institute, Cary, USA). Means and proportions were compared by the student’s *t*-test and Fisher’s exact test, respectively. In stepwise linear regression, we forced age, gender, body weight and body height, and considered systolic and diastolic blood pressure, current smoking and drinking, the use of antihypertensive drugs, plasma glucose, and the total-to-HDL cholesterol ratio as potential confounders of left atrial volume, LVM and E/A ratio. We performed stepwise logistic regression analyses to investigate correlates of left atrial enlargement, left ventricular hypertrophy and diastolic dysfunction in the consideration of above-mentioned variables. In the stepwise regression analysis, *P* < 0.10 were set as criteria for independent variables to enter and stay in the model. We also applied ANOVA to compare the prevalence of cardiac structural and functional abnormalities according to alcohol consumption, plasma glucose, and systolic and diastolic blood pressure.

## Results

The 843 participants included 288 (34.2%) women, 191 (22.7%) hypertensive patients (of whom 82 [42.9%] took antihypertensive drugs), 87 (10.4%) patients with hyperlipidemia, 17 (2.0%) patients with diabetes mellitus, and 2 (0.3%) patients with a LVEF less than 50%. Table 1 shows the characteristics of our study subjects. Men and women had similar age (43.0±14.0 years, range 15 to 79 years), plasma glucose (4.37±1.19 mmol/L) and total cholesterol (4.82±0.92 mmol/L). However, men, compared with women, had a greater body mass index (23.9 ± 3.1 vs 22.5 ± 3.1 kg/m^2^, *P* < 0.001), higher systolic/diastolic blood pressure (124.5 ± 18.3/76.7 ± 11.4 vs 117.5 ± 20.6/72.0 ± 10.6 mmHg, *P* < 0.001), more frequently reported smoking and drinking (46.8% vs 0.7%, 38.4% vs 1.8%, *P* < 0.001), and had lower HDL cholesterol (1.30 ± 0.35 vs 1.50 ± 0.37 mmol/L, *P* < 0.001) and higher LDL cholesterol (3.12 ± 0.83 vs 2.95 ± 0.83 mmol/L, *P* = 0.005) and triglycerides (2.17 ± 1.98 vs 1.57 ± 1.10 mmol/L, *P* < 0.001). All echocardiographic measurements were significantly higher in men than women (*P* ≤ 0.03), but women, compared to men, had significantly higher LVEF and E/A ratio (*P* = 0.001).

**Table 1:** Characteristics of participants

	**Men (n = 555)**	**Women (n = 288)**	***P***
Age, years	43.5 ± 14.7	42.1 ± 12.5	0.15
Body height, cm	168 ± 6	158 ± 5	<0.001
Body weight, kg	67.4 ± 9.9	55.8 ± 8.0	<0.001
Body mass index, kg/m^2^	23.9 ± 3.1	22.5 ± 3.1	<0.001
Body surface area, m^2^	1.80 ± 0.15	1.58 ± 0.11	<0.001
Systolic blood pressure, mmHg	124.5 ± 18.3	117.5 ± 20.6	<0.001
Diastolic blood pressure, mmHg	76.6 ± 11.4	72.0 ± 10.6	<0.001
Current smoking, %	255 (46.8)	2 (0.7)	<0.001
Current drinking, %	209 (38.4)	5 (1.8)	<0.001
Taking antihypertensive drugs, n (%)	61 (11.2)	21 (7.4)	0.08
Plasma glucose, mmol/L	4.37 ± 1.49	4.37 ± 0.75	0.98
Total cholesterol, mmol/L	4.85 ± 0.92	4.76 ± 0.92	0.17
Low-density lipoprotein cholesterol, mmol/L	3.12 ± 0.83	2.95 ± 0.83	0.005
High-density lipoprotein cholesterol, mmol/L	1.30 ± 0.35	1.50 ± 0.37	<0.001
Triglycerides, mmol/L	2.17 ± 1.98	1.57 ± 1.10	<0.001
Left atrial volume index, mL/m^2^	19.3 ± 4.6	18.6 ± 3.8	0.03
Left ventricular mass index, g/m^2^	85.9 ± 16.2	73.3 ± 14.3	<0.001
Relative wall thickness	0.38 ± 0.05	0.35 ± 0.05	<0.001
Left ventricular ejection fraction, %	62.5 ± 4.9	64.0 ± 5.0	<0.001
E/A ratio	1.15 ± 0.38	1.25 ± 0.39	<0.001
Deceleration time, ms	196 ± 28	183 ± 27	<0.001
Left atrial enlargement, n (%)	15 (2.9)	3 (1.2)	0.14
Left ventricular hypertrophy, n (%)	25 (4.5)	17 (5.9)	0.38
Left ventricular diastolic dysfunction, n (%)	95 (20.4)	26 (11.4)	0.003

Data are mean ± standard deviation or number with percentage in parenthesis. E/A ratio indicates the ratio of early transmitral diastolic peak flow (E) and atrial peak flow (A). Deceleration time indicates the deceleration time of early transmitral diastolic flow (E). For the definition of left atrial enlargement, left ventricular hypertrophy and diastolic dysfunction, see Methods.

In continuous analyses, in addition to age, male gender, body height and body weight, left atrial volume was significantly associated with systolic blood pressure (*P* < 0.001) and plasma glucose (*P* = 0.008), LVM with systolic blood pressure (*P* < 0.001), and E/A ratio with diastolic blood pressure (*P* < 0.001) and the total-to-HDL cholesterol ratio (*P* = 0.003, Table 2).

**Table 2:** Cardiac structure and function in relation to conventional cardiovascular risk factors

	**Left atrial volume, ml**			**Left ventricular mass, g**			**E/A ratio**		
	**β ± SE**	**Partial R**^**2**^	***p***	**β ± SE**	**Partial R**^**2**^	***p***	**β ± SE**	**Partial R**^**2**^	***p***
Age, (+10 years)	0.79 ± 0.22	0.10	<0.001	1.09 ± 0.82	0.03	0.18	-0.16 ± 0.01	0.46	<0.001
Gender, (1 = man, 0 = woman)	0.24 ± 0.71	0.07	0.73	23.15 ± 2.65	0.28	<0.001	-0.03 ± 0.03	0.01	0.31
Body height, (+10 cm)	-1.10 ± 0.51	0.03	0.03	2.45 ± 1.94	0.03	0.21	1.39 ± 1.95	0.01	0.47
Body weight, kg	0.49 ± 0.03	0.22	<0.001	1.07 ± 0.13	0.07	<0.001	-0.02 ± 0.01	0.02	0.21
Systolic blood pressure, (+10 mmHg)	0.59 ± 0.17	0.01	<0.001	2.65 ± 0.61	0.01	<0.001	–	–	–
Plasma glucose, mmol/L	0.51 ± 0.19	0.01	0.008	–	–	–	–	–	–
Diastolic blood pressure, (+10 mmHg)	–	–	–	–	–	–	-0.07 ± 0.01	0.03	<0.001
Total-to-HDL cholesterol ratio	–	–	–	–	–	–	-0.03 ± 0.01	0.01	0.003

In stepwise linear regression, we forced age, gender, body weight and body height in the model, and considered systolic and diastolic blood pressure, current smoking and drinking, the use of antihypertensive drugs, plasma glucose, and total-to-HDL cholesterol ratio as potential confounders. *P* value for variables to enter or stay in the model was set at <0.10. β, estimated parameter; SE, standard error; HDL, high density lipoprotein.

**Figure 1:**
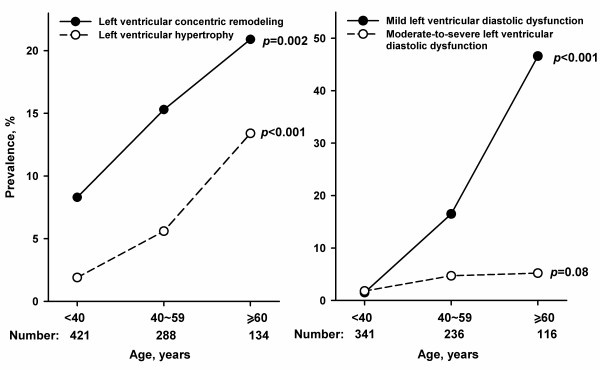
**Prevalence of left ventricular structural and functional abnormalities by age.** On the left panel, left ventricular concentric remodeling and hypertrophy were presented as filled circles with solid line, and open circles with dashed line, respectively. On the right panel, mild and moderate-to-severe left ventricular diastolic dysfunction was presented as filled circles with solid line, and open circles with dashed line, respectively. The number of subjects for each subgroup is given at bottom, and *P* for trend for each line at the right side.

In 843 participants, the prevalence of left atrial enlargement, and left ventricular concentric remodeling and hypertrophy was 2.4%, 12.7% and 5.0%, respectively. In 693 subjects with sufficient echocardiographic measurements to evaluate left ventricular diastolic function, the prevalence of left ventricular diastolic dysfunction was 17.5%, with mild and moderate-to-severe stages of 14.2% and 3.3%, respectively. The prevalence of these cardiac structural and functional abnormalities significantly increased with age, except moderate-to-severe left ventricular diastolic dysfunction (*P* = 0.08). Indeed, the prevalence of left ventricular concentric remodeling increased from 8.3% in subjects younger than 40 years (n = 421) to 20.9% in subjects of at least 60 years (n = 134). The corresponding values were from 1.9% to 13.4% for left ventricular hypertrophy, and from 1.5% to 46.6% for left ventricular diastolic dysfunction (Figure 1).

In addition to age, male gender, body height and body weight, left atrial enlargement was significantly associated with plasma glucose (odds ratio [OR] = 1.29, 95% confidential interval [CI]: 1.06-1.55), left ventricular hypertrophy with systolic blood pressure (OR = 1.21, 95% CI: 1.02-1.44), and left ventricular diastolic dysfunction with diastolic blood pressure (OR = 1.29, 95% CI: 1.03-1.62, Table 3). In addition, the prevalence of left atrial enlargement tended to be higher in drinkers than non-drinkers (OR = 3.16, 95% CI: 0.88-11.33, *P* = 0.08).

**Table 3:** Cardiac structural and functional abnormalities in relation to conventional cardiovascular risk factors

	Left atrial enlargement *			Left ventricular hypertrophy *			Left ventricular diastolic dysfunction *		
	**Odds ratio**	**95% CI**	***p***	**Odds ratio**	**95% CI**	***p***	**Odds ratio**	**95% CI**	***p***
Age, (+10 years)	2.67	1.45-4.93	0.002	1.49	1.11-2.01	0.009	3.08	2.40-3.95	<0.001
Gender, (1 = man, 0 = woman)	0.31	0.05-1.94	0.21	0.78	0.36-2.17	0.78	0.87	0.44-1.74	0.70
Body height, (+10 cm)	1.09	0.37-3.20	0.88	0.72	0.36-1.44	0.35	1.04	0.64-1.69	0.88
Body weight, kg	1.10	1.03-1.18	0.003	1.02	0.98-1.07	0.31	1.06	1.02-1.09	<0.001
Current drinking, (1 = yes, 0 = no)	3.16	0.88-11.33	0.08	–	–	–	–	–	–
Plasma glucose, mmol/L	1.29	1.06-1.55	0.009	–	–	–	–	–	–
Systolic blood pressure, (+10 mmHg)	–	–	–	1.21	1.02-1.44	0.03	–	–	–
Diastolic blood pressure, (+10 mmHg)	–	–	–	–	–	–	1.29	1.03-1.62	0.02

* Multinomial logistic regression was applied for left atrial enlargement and left ventricular hypertrophy; and multinomial ordinal logistic regression for left ventricular diastolic dysfunction with 0 = normal, 1 = mild diastolic dysfunction, and 2 = moderate or severe diastolic dysfunction. In stepwise logistic regression, we forced age, gender, body weight and body height in the model, and considered systolic and diastolic blood pressure, current smoking and drinking, the use of antihypertensive drugs, plasma glucose, and total-to-HDL cholesterol ratio as potential confounders. *P* value for variables to enter or stay in the model was set at <0.10. CI, confidential interval.

**Figure 2:**
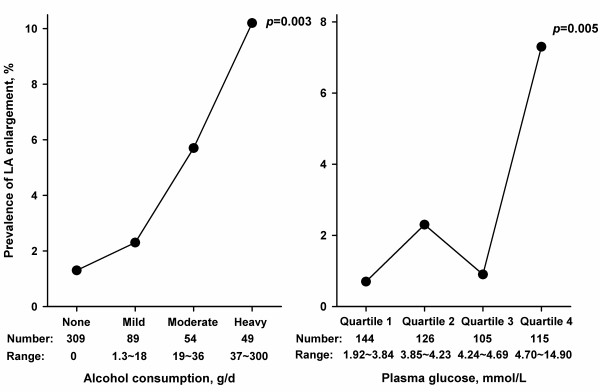
**Prevalence of left atrial enlargement in relation to alcohol consumption and plasma glucose in men.** Subjects were classified by tertiles of daily alcohol consumption (on the left panel), with non-drinker, and mild, moderate and heavy drinking defined as daily alcohol consumption = 0 g/d, <18 g/d, 18 to 36 g/d, and > 36 g/d, respectively, and classified by quartiles of plasma glucose (on the right panel). The number of subjects and the range of values for each subgroup are given at bottom, and *P* for trend for each line at the right side.

**Figure 3:**
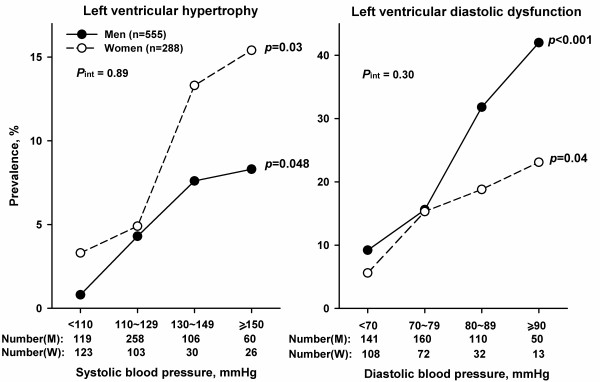
**Prevalence of left ventricular hypertrophy and diastolic dysfunction in relation to systolic and diastolic blood pressure.** The number of subjects for each subgroup is given at bottom, and *P* for trend for each line at the right side.

In further analyses, we studied left atrial enlargement in men (too small numbers in women) and left ventricular hypertrophy and diastolic dysfunction in men and women, separately, in relation to their correlates identified from above-mentioned analysis as categorical variables. The prevalence of left atrial enlargement in men increased from 1.29% in non-drinkers (n = 309), to 2.25% in mild drinkers (n = 89), to 5.56% in moderate drinkers (n = 54), and to 10.2% in heavy drinkers (n = 49, *P* = 0.003 for trend), and remained at low level (1.3%) in subjects with a plasma glucose concentration below 4.70 mmol/L (quartiles 1 to 3) and increased to 7.3% in subjects with plasma glucose concentration above 4.70 mmol/L (quartile 4, Figure 2). The prevalence of left ventricular hypertrophy and diastolic dysfunction increased with higher levels of systolic and diastolic blood pressure, respectively, but with slightly steeper slope in women for hypertrophy (*P* for interaction = 0.89) and in men for diastolic dysfunction (*P* for interaction = 0.30, Figure 3).

## Discussion

To our knowledge, our study is the first that has reported the prevalence and correlates of the echocardiographically diagnosed left atrial enlargement, left ventricular hypertrophy and diastolic dysfunction in an epidemiological setting in a Chinese population. In our study, the prevalence of left atrial enlargement, left ventricular hypertrophy and diastolic dysfunction was 2.4%, 5.0% and 17.5% respectively. The prevalence of these cardiac structural and functional abnormalities increased with age, and for left atrial enlargement also with alcohol consumption and plasma glucose, particularly in men, and for left ventricular hypertrophy and diastolic dysfunction, respectively, also with systolic and diastolic blood pressure.

Our finding is comparable with the results of a recent Vietnamese study [17] and the Strong Heart Study in American Indians for left ventricular hypertrophy [18], and with the results of the Olmsted County studies in the Americans [19] and the MONICA study in Germans [20] for left ventricular diastolic dysfunction. In 508 Vietnamese, using similar diagnostic technique as ours, the prevalence of left ventricular hypertrophy was 8.5% [17]. In 2400 participants of the Strong Heart Study, the overall prevalence of left ventricular hypertrophy was 6.8% [18]. In 2042 American residents of Olmsted County, the prevalence of mild, moderate and severe left ventricular diastolic dysfunction was 20.8%, 6.6% and 0.7%, respectively, and significantly increased with age [19]. In 1274 Germans in the MONICA study, the prevalence of mild and moderate-to-severe left ventricular diastolic dysfunction was 11.1% and 3.1%, respectively [20].

There is clear and solid evidence that left ventricular hypertrophy was significantly associated with increased systolic blood pressure. We found a significant and linear association between left ventricular diastolic dysfunction and diastolic blood pressure, with a slightly steeper slope in men than in women. This association could be explained by many possibilities. Peripheral resistance is a major determinant of diastolic blood pressure. When vascular remodeling in arterioles is developed, peripheral resistance increases and coronary flow reserve decreases, which in the long run may cause myocardial ischemia, fibrosis, and diastolic dysfunction, before systolic dysfunction develops. Thus, it is possible that arterial remodeling is the common cause of diastolic dysfunction and high diastolic blood pressure. One recent study might lend indirect support for this explanation [21]. In 233 subjects, diastolic dysfunction was significantly associated with increased augmentation index, which is a hallmark of peripheral remodeling and arterial stiffening. Of course, the cross-sectional design of our study does not allow any causal inference.

Our finding regarding the association between left atrial enlargement and alcohol consumption is in line with the results of two previous studies [22,23]. In 1354 subjects of the HyperGEN study [22], it was reported that left atrial size was positively and significantly associated with alcohol consumption. Similarly, in a relatively smaller study (n = 48), left atrial size significantly decreased in chronic alcoholics after a 12-day abstinence [23]. Further studies, especially in a prospective setting, are apparently warranted.

It is unexpected to find a significant association of left atrial volume and enlargement with plasma glucose in the present study. In literature, Milutinović S et al. have reported that hypertensive patients with diabetes, compared to those without, had significantly higher left atrial volume [24]. We found in our study that left atrial enlargement was significantly related to plasma glucose in a nonlinear mode. Patients in the top quartile of plasma glucose had high prevalence of left atrial enlargement (7.2%), which contributed mostly to the observed significant association. Furthermore, in a subgroup analysis in patients without diabetes (n = 813), the associations of left atrial volume index and enlargement with plasma glucose were not statistically significant (*P* ≥ 0.07).

One of the major limitations in the present study is its small sample size, especially in women. In addition, since the present study was conducted in a relatively young and healthy Chinese population, we applied a “low” criterion to define left ventricular hypertrophy, which was also recommended by ASE. If we applied the criterion of LVMI >125 g/m^2^ (men) or >110 g/m^2^ (women) in the present study, our major finding remained unaltered. Specifically, the prevalence of left ventricular hypertrophy would be 1.7% (n = 14), and its influential factors would include only systolic blood pressure (OR = 1.04 [95%CI: 1.01-1.06], P = 0.005). Secondly, diastolic dysfunction was solely evaluated by Pulse-wave Doppler Imaging, but not Tissue Doppler Imaging in the present study. This technological limitation might, to some extent*,* influence the accuracy in the classification of diastolic dysfunction. However, this conventional diagnostic technique has been used in most of pervious population studies, and is more applicable in the screening for asymptomatic disease. Lastly, since the present population was recruited in a factory located in southeast China, our finding probably cannot be extrapolated to the entire Chinese population.

## Conclusions

In conclusion, even in a middle-aged and relatively healthy Chinese population, cardiac structural and functional abnormalities, mostly asymptomatic, were prevalent, and had higher prevalence with age advancing and in the presence of several known cardiovascular risk factors, such as heavy drinking of alcohol, hyperglycemia, and high blood pressure. The clinical implications of our finding is that echocardiography probably should be performed for screening subclinical cardiac structural and functional abnormalities, especially in the elderly or in the presence of common cardiovascular risk factors.

## Abbreviations

HDL: High-density lipoprotein; LDL: Low-density lipoprotein; ASE: American Society of Echocardiography; LVM: Left ventricular mass; LVMI: Left ventricular mass index; RWT: Relative wall thickness; E/A ratio: Ratio of early transmitral diastolic peak flow (E) and atrial peak flow (A); DTE: Deceleration time of early transmitral diastolic flow; LVEF: Left ventricular ejection fraction; OR: Odds ratio; CI: Confidential interval.

## Competing interests

The authors declare that they have no competing interests.

## Authors’ contributions

YZ has made substantial contributions to the conception and design of the paper and the analysis and interpretation of data, and been involved in drafting the manuscript. YL, ML, CSS and QFH have participated in patient evaluations, data collection, and the analysis of data. JGW has made substantial contributions to the conception and design of the paper, and been involved in revising the manuscript. All the authors have read and approved the final version of the manuscript.

## Pre-publication history

The pre-publication history for this paper can be accessed here:

http://www.biomedcentral.com/1471-2261/12/86/prepub
